# Hygiene and Materia Medica

**Published:** 1873-04

**Authors:** 


### *Hygiene and Materia Medica.*

In a recently published note, the eminent surgeon and teacher, Willard Parker, M.D., uses these words:

"Heretofore, *materia medica* has almost monopolized the attention in our schools of medicine, as if that were the all important agent in the cure of disease."

Against which proposition we earnestly protest as doing gross injustice to many, if not the majority, of the schools. The attention of Prof. Parker to the duties of his extensive practice, and those of his particular department of teaching, has probably prevented his notice of the fact that for a long time, PHYSIOLOGY and its necessary inferential teachings, have taken the leading position in the colleges. Educated medical men, for years, have been investigating the relations of facts, and seeking the laws. Mere medicine seekers and givers have fallen into something like contempt—not for the paltry reason suggested by Professor Parker in these words:

"During the last forty years a form of practice has been introduced which has demonstrated the little value of *medicine alone*, and the public are now demanding more knowledge upon the laws of life and health."

Theoretically and practically, Homœopathy deals constantly and largely in medicine giving. They have specifics for every ill that flesh is heir to, and their *public* believe homœopathic medicines are really "potencies." Neither the "public" nor the profession are under obligations to Homœopathy for their greater concern about sanitary laws.

Medical science advances, and included sanitary science advances, because its study is more and more approximating the methods whereby other sciences are advanced. The *asinus portaus mysteria*, whether ridden by Galen or Hahnemann, has been sent to the rear, even of the baggage-wagons, of the grand army of progress.

The notion of the entity of disease still infects some minds, leading them to hunt for new specifics and panaceas, but the thinking men, the inquiring men, the experimenting men, are wresting from Nature the secrets of Health and Disease, just as Huxley, Tyndall and Agassiz are seizing the grand truths of physical science.

It is sincerely to be hoped that Prof. Parker and those who entertain similar opinions may look over the facts a little more closely, before affording any more such "hasty plates of soup" for the delectation of the infinitesimal crew.

